# Nuclear envelope localization of PIG-B is essential for GPI-anchor synthesis in *Drosophila*

**DOI:** 10.1242/jcs.218024

**Published:** 2018-10-26

**Authors:** Miki Yamamoto-Hino, Eri Katsumata, Emiko Suzuki, Yusuke Maeda, Taroh Kinoshita, Satoshi Goto

**Affiliations:** 1 Department of Life Science, Rikkyo University, Toshima-ku, Tokyo 171-8501, Japan; 2 Gene Network Laboratory, National Institute of Genetics, Mishima, Shizuoka 411-8540, Japan; 3 Research Institute for Microbial Diseases, Osaka University, Suita, Osaka 565-0871, Japan

**Keywords:** Glycosylphosphatidylinositol, Nuclear envelope, Endoplasmic reticulum, PIG, Quality control, Functional organelle zones, *Drosophila*

## Abstract

Membrane lipid biosynthesis is a complex process that takes place in various intracellular compartments. Glycosylphosphatidylinositol (GPI), a lipid involved in membrane anchoring of some proteins, is synthesized by the PIG enzymes. Most PIGs are localized to the endoplasmic reticulum (ER), but *Drosophila* PIG-B (DmPIG-B) is localized to the nuclear envelope (NE). To determine whether the NE localization of DmPIG-B is functionally important, we defined the determinants of localization and generated an ER-localized form, denoted DmPIG-B[ER]. The enzymatic activity of DmPIG-B[ER] was comparable to that of NE-localized DmPIG-B[NE]. Expression of DmPIG-B[ER] inefficiently rescued the lethality of the *PIG-B* mutant, whereas DmPIG-B[NE] rescued this lethality fully. DmPIG-B[ER] was preferentially degraded by lysosomes, suggesting that the NE localization is essential for function and stability of the protein. In addition, we found that the region of the ER proximal to the NE is the site of translation of GPI-anchored proteins and addition of GPI. Thus, the NE and proximal ER may provide a platform for efficient GPI anchoring.

## Abstract (precis)

**Summary:** In *Drosophila*, localization of the enzyme PIG-B in the nuclear envelope (NE) is important for GPI anchor synthesis, and the NE and the perinuclear ER form a platform for the GPI modification.

## INTRODUCTION

Membrane lipid metabolism takes place in various cellular compartments, including the endoplasmic reticulum (ER), mitochondria, Golgi and endosomes. The lipid glycosylphosphatidylinositol (GPI) is used as a membrane anchor for multiple cellular proteins. GPI-anchored proteins play important roles in development, immunity, neural functions and physiological homeostasis. In humans, defects in GPI-anchor formation cause diseases such as paroxysmal nocturnal hemoglobinuria and inherited GPI deficiency (Almeida et al., 2009; Nishimura et al., 1999).

**Fig. 1.**
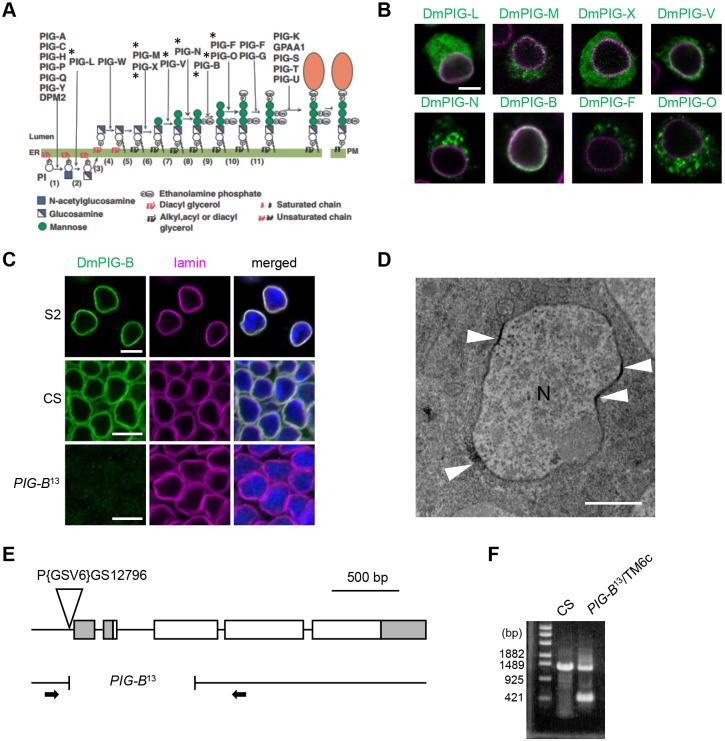
**Subcellular localization of proteins involved in GPI-anchor synthesis.** (A) Reactions and enzymes involved in mammalian GPI-anchor synthesis. Asterisks indicate enzymes for which subcellular localization has been studied in *Drosophila* S2 cells. (B) Myc-tagged DmPIG proteins stained with anti-Myc antibody (green) and NE labeled with anti-lamin antibody (magenta). Scale bar: 5 µm. (C) NE localization of endogenous DmPIG-B (green) in S2 cells (S2) and wing disc cells of the wild type (CS) and the *PIG-B* mutant (*PIG-B^13^*). NE and nucleus were labeled with anti-lamin antibody (magenta) and DAPI (blue), respectively. Scale bars: 5 µm. (D) Immunoelectron microscope image of DmPIG-B in wing disc cells. DmPIG-B was detected at or very near a part of the nuclear membrane (arrowheads). N, Nucleus. Scale bar: 1 µm. (E) Map of the *PIG-B* locus showing the P{GSV6}GS12796 insertion and the breakpoints of the *PIG-B*^13^ deletion allele. White and gray boxes represent DmPIG-B protein-coding and non-coding regions, respectively. Arrows indicate primers used in F. (F) PCR fragments amplified from wild-type (CS) and *PIG-B*^13^ genomes. Breakpoints in *PIG-B*^13^ were determined by sequencing of the PCR fragment.

GPI is synthesized from phosphatidylinositol in a multi-step reaction catalyzed by PIG enzymes (Kinoshita and Fujita, 2016). To date, 17 enzymes have been identified in mammals (Fig. 1A) (Kinoshita, 2014). Among them, PIG-B catalyzes addition of the third mannose, a later step in GPI synthesis (Takahashi et al., 1996). This enzyme was identified by expression screening of cDNAs using class B Thy-1-deficient murine T-lymphoma cells, in which the transfer of Man-3 is defective. Thy-1 is a GPI-anchored protein and is therefore not present on the surface of class B cells. Transfection of PIG-B cDNA restores Thy-1 expression on the surface of class B cells.

Exogenously expressed PIG enzymes localize throughout the ER in cultured mammalian cells (Kang et al., 2005; Maeda et al., 2001; Murakami et al., 2005, 2003; Nakamura et al., 1997; Taron et al., 2004; Vainauskas et al., 2002; Watanabe et al., 1996). However, some endogenous enzymatic activities involved in GPI synthesis are specifically observed in a subcompartment of the ER (Vidugiriene et al., 1999).

GPI is transferred to proteins by a transamidase complex (TAC) consisting of PIG-T, PIG-K, PIG-U, PIG-S and GPAA1, yielding GPI-anchored proteins (Hong et al., 2003; Ohishi et al., 2001). Subsequently, the GPI moiety is remodeled by additional enzymes. In yeast, remodeled GPI-anchored proteins are recognized by the p24 complex and loaded into transport vesicles distinct from those containing other transmembrane proteins (Castillon et al., 2011; Muñiz et al., 2001; Muñiz and Riezman, 2016). Therefore, GPI synthesis, GPI anchoring of proteins and loading into transport vesicles may take place in a subcompartment of the ER.

Here, we show that *Drosophila* PIG-B (DmPIG-B) is localized to the nuclear envelope (NE), whereas other PIG enzymes are localized to the ER. To determine whether the NE localization of DmPIG-B is essential for GPI production, we generated an ER-localized DmPIG-B, DmPIG-B[ER], which has activity comparable to that of the wild-type protein, NE-localized DmPIG-B[NE]. Expression of DmPIG-B[ER] did not efficiently rescue the lethality of the *PIG-B*-null mutant, whereas DmPIG-B[NE] rescued the lethality almost completely. The level of DmPIG-B[ER] protein, but not that of the corresponding mRNA, was diminished in comparison with that of DmPIG-B[NE]. To compare the rescue efficiencies of DmPIG-B[ER] and DmPIG-B[NE] when they are expressed at comparable levels, we reduced expression of DmPIG-B[NE] by culturing flies at 18°C. The rescue efficiency of DmPIG-B[NE] was significantly higher than that of DmPIG-B[ER]. These results suggest that NE localization is essential for the function and stability of DmPIG-B. In addition, translation and GPI addition of GPI-anchored proteins took place in the NE and at ER that was proximal to the NE. Thus, the NE and proximal ER provide a functional platform for efficient formation of GPI-anchored proteins by ensuring close proximity of the later steps of GPI synthesis and translation, and GPI anchoring.

## RESULTS

### DmPIG-B localizes to the NE

Seventeen enzymes involved in synthesis of GPI from phosphatidylinositol have been identified in mammals (Fig. 1A) (Kinoshita, 2014). When these enzymes are overexpressed, they localize to the ER, suggesting that GPI is synthesized entirely in the ER. To determine whether all steps in GPI synthesis in *Drosophila* occur in the ER, we overexpressed Myc-tagged versions of *Drosophila* PIG orthologs (DmPIGs) in *Drosophila* S2 cells. Immunostaining with anti-Myc antibody revealed that only DmPIG-B colocalized with lamin, a major component of the nuclear lamina (Fig. 1B), indicating that it resided in the NE. By contrast, DmPIG-L, -M, -X and -V were detected in cytoplasmic regions, possibly including the ER (Fig. 1B). DmPIG-N, -F and -O localized to round structures that appeared to differ from those that DmPIG-L, -M, -X and -V localized to. Therefore, we performed co-staining for the ER marker calreticulin (Calr)-GFP and the late endosomal marker Rab7 (Fig. S1). DmPIG-N, -F and -O were localized to the ER, but did not colocalize with Rab7, with the exception of a small pool of DmPIG-O. Overexpression of DmPIG-O may cause it to overspill from the ER to late endosomes.

To confirm the NE localization of endogenous DmPIG-B, we generated an anti-DmPIG-B antibody. Immunofluorescence analysis of S2 cells and wing discs [S2 and CS (*Canton-S*), respectively, in Fig. 1C] and immunoelectron microscopic analysis of wing discs (Fig. 1D) showed that endogenous DmPIG-B protein was localized to the NE. The signals detected by the anti-DmPIG-B antibody were not detected in wing disc cells from a *PIG-B*-null mutant (*PIG-B*^13^, Fig. 1C), which harbors a 938 bp deletion within the *PIG-B* gene that removes the first ATG (Fig. 1E,F); the mutant larvae die at the late larval stage. Thus, DmPIG-B protein is localized to the NE.

### DmPIG-B is involved in GPI synthesis

**Fig. 2.**
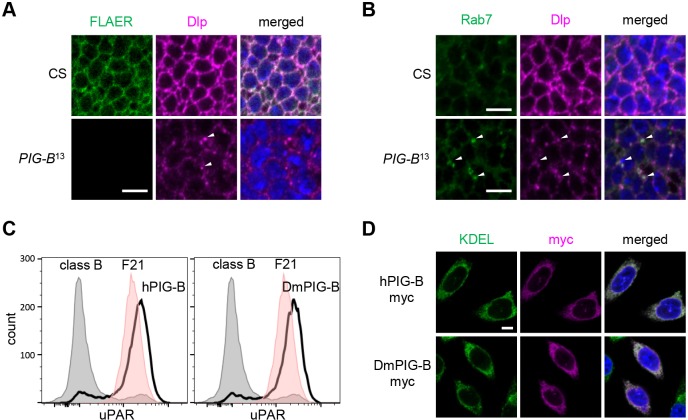
**DmPIG-B is essential for GPI synthesis and cell-surface expression of GPI-anchored proteins.** (A) Localization of GPI-anchored proteins in wing disc cells of CS (control) and *PIG-B*^13^ mutant. GPI-anchor was labeled with FLAER (green). Dlp and nuclear DNA are shown in magenta and blue, respectively. Arrowheads indicate accumulation of Dlp in the cytosol. Scale bar: 5 µm. (B) In *PIG-B*^13^, arrowheads indicate accumulation of Dlp (magenta) in Rab7-positive late endosomes (green). Scale bars: 5 µm. (C) Restoration of surface expression of uPAR on class B mutant CHO cells upon transfection with Myc-tagged human (left panel) and *Drosophila* (right panel) PIG-B cDNA as determined by flow cytometry. Gray, class B cells; red, parental cell line F21; bold black line, class B cells expressing Myc-tagged PIG-B. (D) Immunofluorescence analysis of CHO cells expressing Myc-tagged human (upper panels) and *Drosophila* (lower panels) PIG-B (magenta). ER and nuclear DNA were labeled with anti-KDEL (green) antibody and DAPI (blue), respectively. Scale bar: 5 µm.

We next investigated whether DmPIG-B is involved in GPI synthesis. First, we detected GPI moieties in wing discs of *PIG-B*^13^ and control (CS) larvae using fluorescently labeled inactive toxin aerolysin (FLAER) (Brodsky et al., 2000). In controls, GPI-anchored proteins were expressed on the cell surface, resulting in fluorescent signals along cell outlines (Fig. 2A). However, no fluorescence was detected in wing discs of *PIG-B*^13^. Second, we observed the localization of Dally-like protein (Dlp), a GPI-anchored protein expressed in wing discs (Baeg et al., 2001). Dlp was located at the cell surface in wild-type wing discs, but accumulated in Rab7-positive late endosomes in *PIG-B*^13^ mutants (Fig. 2A,B). Therefore, DmPIG-B is required for GPI production and cell surface expression of GPI-anchored proteins.

Human PIG-B (hPIG-B) adds the third mannose of GPI (Fig. 1A). We assayed DmPIG-B mannosyltransferase activity by testing whether it could restore cell surface expression of a GPI-anchor protein in PIG-B-deficient CHO cells (class B cells). Urokinase plasminogen activator receptor (uPAR) was expressed on the surface of F21 cells (the parent of class B cells) (Maeda et al., 2006), but not in class B cells (Fig. 2C). When Myc-tagged hPIG-B or DmPIG-B was expressed in class B cells, surface expression of uPAR was fully restored in both cases. The expressed hPIG-B and DmPIG-B colocalized with staining for proteins containing a KDEL sequence, marking the ER (Fig. 2D), indicating that both variants of PIG-B were located in the ER. Thus, DmPIG-B has the ability to add the third mannose of GPI.

### DmPIG-B has interspersed regions needed for the NE localization

DmPIG-B is localized to the NE in *Drosophila*, whereas other DmPIGs reside in the ER (Fig. 1B). To determine whether NE localization is essential for DmPIG-B function, we defined the regions required for NE localization in DmPIG-B, and then altered amino acid sequences in these NE localization determinant sequences to generate an ER-localized form of DmPIG-B.

**Fig. 3.**
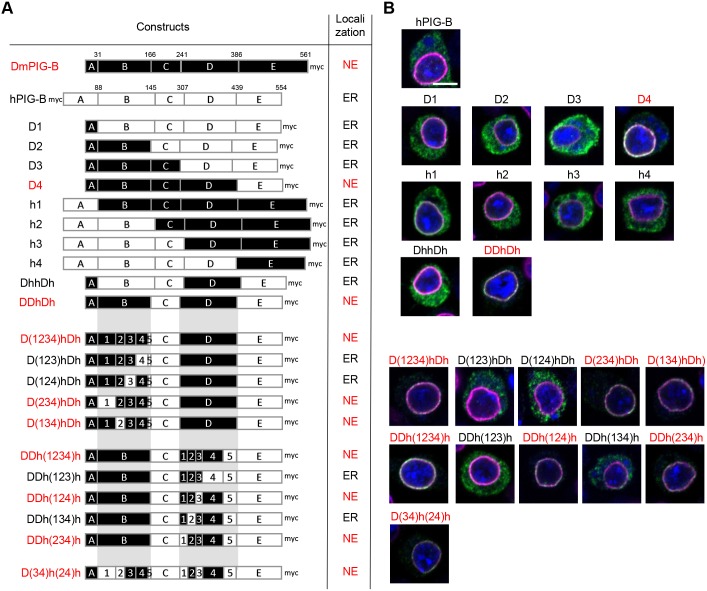
**Regions required for NE localization of DmPIG-B.** (A) Schematic representation and localization of native *Drosophila*, human and chimeric PIG-B proteins. Segments derived from DmPIG-B and hPIG-B are shown as black and white boxes, respectively. Numbers above boxes indicate the amino acid positions of DmPIG-B and hPIG-B in each segment. Red characters indicate the NE localization of PIG-B protein as shown in B. (B) Immunofluorescence analysis of S2 cells expressing Myc-tagged human and chimeric PIG-B proteins. Cells were stained with anti-Myc (green) and anti-lamin (magenta) antibodies, and DAPI (blue). Scale bar: 5 µm.

**Fig. 4.**
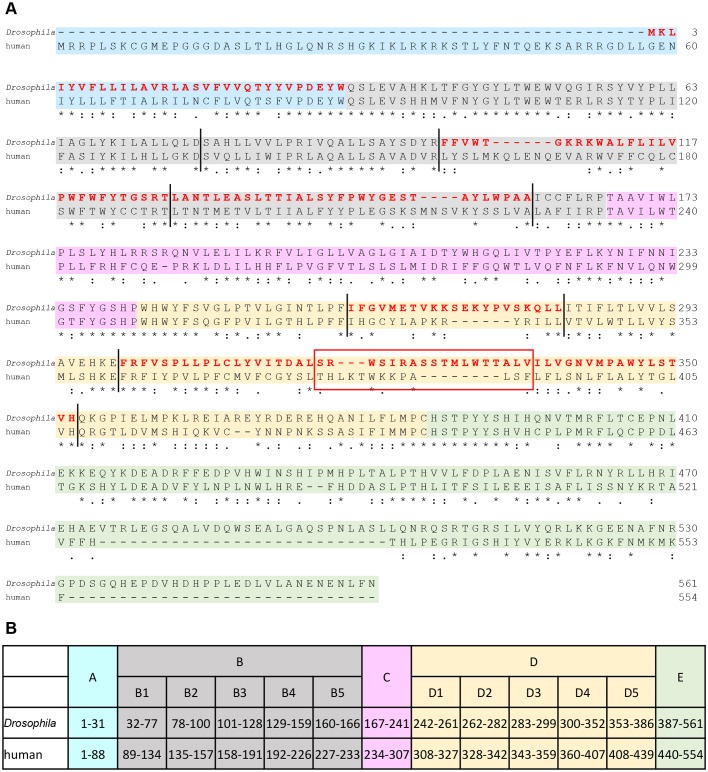
**Alignment of *Drosophila* and human PIG-B amino acid sequences.** (A) *Drosophila* and human PIG-B amino acid sequences were aligned using ClustalW. Symbols: asterisks, identical amino acids at the same position in *Drosophila* and human PIG-B; colons, similar amino acids at the same position in *Drosophila* and human PIG-B. The background color indicates the segments of PIG-B mentioned in Fig. 3A (blue, segment A; gray, segment B; pink, segment C; yellow, segment D; green, segment E). Red highlighted characters show the amino acid sequences needed for NE localization of DmPIG-B. The red box indicates the region in which sequence was exchanged between *Drosophila* and human PIG-B to generate DmPIG-B[ER]5 (Fig. 5). Vertical lines indicate positions swapped between *Drosophila* and human PIG-B to analyze the subsegments of B and D. (B) Amino acid positions of each segment in *Drosophila* and human PIG-B in Fig. 3A.

Myc-tagged hPIG-B localized to the ER in S2 cells, although its expression level was very low (Fig. 3A,B), suggesting that DmPIG-B contains NE localization signals, whereas hPIG-B does not. To determine the regions responsible for NE localization in DmPIG-B, we aligned the amino acid sequences of DmPIG-B and hPIG-B, divided into five segments (Fig. 4), and constructed a series of chimeric proteins (Fig. 3A). Only chimeric protein D4 was localized to the NE (Fig. 3B), suggesting that segments A and D are necessary for NE localization. However, chimeric protein DhhDh failed to localize to the NE. Additional analysis revealed that DDhDh localized to the NE, indicating that the A, B and D segments are necessary and sufficient for NE localization.

**Fig. 5.**
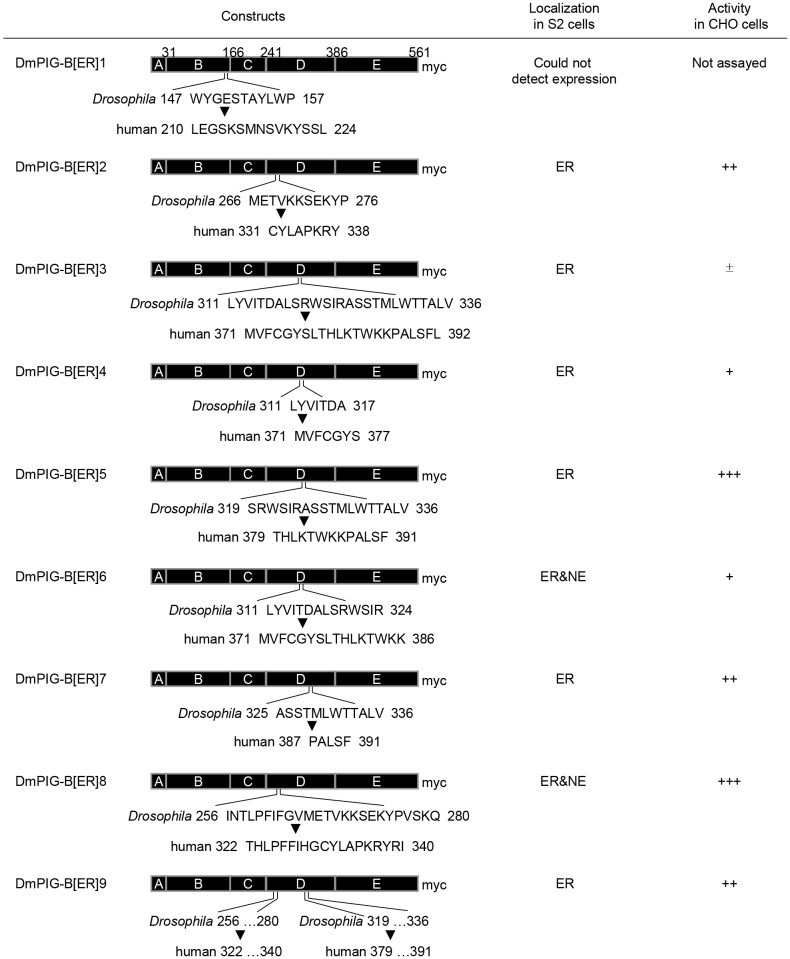
**Schematic representation of chimeric ER-localized DmPIG-B variants constructed in this study.** Chimeric DmPIG-B variants, their localizations in S2 cells and their abilities to restore the uPAR cell surface expression in CHO class B cells are presented. +++, full activity; ++, half activity; +, weak activity; ±, no activity.

Next, we precisely defined the portions of the B and D segments necessary for NE localization. To this end, we divided those two segments into five subsegments, and again constructed chimeric molecules (Figs 3A and 4). All NE-localized chimeric proteins had *Drosophila* B3 and B4 subsegments, suggesting that both were needed for NE localization. Similarly, the *Drosophila* D2 and D4 subsegments were also required for NE localization. Collectively, amino acids (aa) 1–31 (segment A), 101–159 (subsegments B3 and B4), 262–282 (subsegment D2) and 300–352 (subsegment D4) were required for NE localization of DmPIG-B (Fig. 4B). To confirm that these sequences were sufficient for NE localization, we constructed the D(34)h(24)h chimeric protein and transfected it into S2 cells (Fig. 3A). Immunofluorescence analysis revealed that the chimeric protein mainly localized to the NE (Fig. 3B), indicating that these regions are necessary and sufficient for NE localization of DmPIG-B.

### NE localization of DmPIG-B is essential for its function and stability

**Fig. 6.**
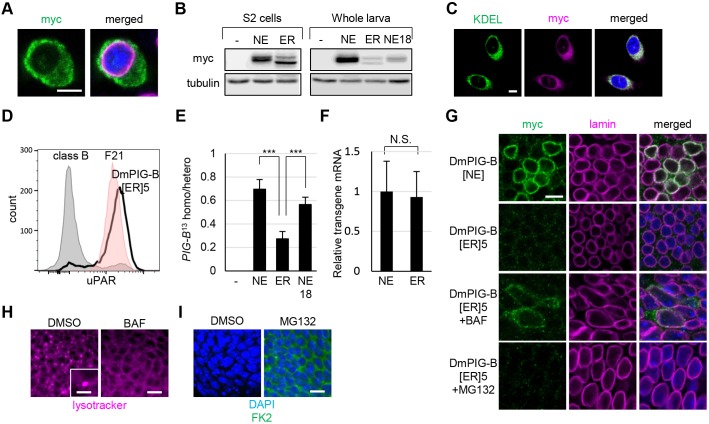
**ER-localized DmPIG-B does not rescue lethality in *PIG-B*^13^ and is degraded by a lysosomal function.** (A) Immunofluorescence analysis of S2 cells expressing DmPIG-B[ER]5. Cells were stained with anti-Myc (green) and anti-lamin (magenta) antibodies, and DAPI (blue). Scale bar: 5 µm. (B) Immunoblot analysis of Myc-tagged DmPIG-B[NE] (NE) and DmPIG-B[ER]5 (ER) expressed in S2 cells (left) or in third-instar larvae under the control of da-Gal4 at 25°C (NE, ER) or 18°C (NE18) (right). Proteins were detected with anti-Myc and anti-tubulin (loading control) antibodies. The relative signal strength (Myc/tubulin) in whole larvae was: NE, 5.79; ER, 1.19; NE18, 0.90. (C) Immunofluorescence analysis of CHO cells expressing DmPIG-B[ER]5. Cells were stained with anti-KDEL (green) and anti-Myc (magenta) antibodies, and DAPI (blue). Scale bar: 5 µm. (D) Restoration of surface expression of uPAR on class B mutant CHO cells by transfection with DmPIG-B[ER]5 cDNA. Gray, class B cells; red, parental cell line F21; bold black line, class B cells expressing DmPIG-B[ER]5. (E) Rescue of *PIG-B*^13^ lethality by expression of Myc-tagged DmPIG-B[NE] (NE) and DmPIG-B[ER]5 (ER) under the control of da-Gal4 at 25°C (NE, ER) or 18°C (NE18). Values are means±s.d. of data obtained in three independent crosses; more than 100 adults were scored for each cross. ***P<0.005 as determined by Student's *t*-test. (F) mRNA levels of Myc-tagged DmPIG-B[NE] (NE) and DmPIG-B[ER]5 (ER) expressed in third-instar larvae under the control of da-Gal4 (means±s.d., *n*=30). N.S., not significant as determined by Student's *t*-test. (G) Immunofluorescence analysis of wing discs expressing Myc-tagged DmPIG-B[NE] and DmPIG-B[ER]5 under the control of da-Gal4, following bafilomycin (BAF) or MG132 treatment. Wing discs were stained with anti-Myc (green) and anti-lamin (magenta) antibodies, and DAPI (blue). Scale bar: 5 µm. (H) LysoTracker Red staining (magenta) of wing discs treated with BAF or DMSO. The inset in the DMSO-treated panel shows a LysoTracker Red-positive vesicle, which was not observed in BAF-treated wing discs. Scale bar: 5 µm (main image); 2.5 µm (inset). (I) Immunofluorescence analysis of wing discs treated with MG132 or DMSO. Wing discs were stained with the anti-ubiquitin antibody FK2 (green) and DAPI (blue). Scale bar: 5 µm.

DmPIG-B is the only GPI synthesis enzyme residing in the NE. To address the significance of this localization, we asked whether ER-localized DmPIG-B could rescue the lethality of *PIG-B*^13^. We first tried to express Myc-tagged hPIG-B in *PIG-B*^13^, but the expression level and rescue efficiency were very low (Fig. S2), raising the possibility that hPIG-B is unstable in *Drosophila*. Differences in codon usage between human and *Drosophila* may influence the translation efficiency; therefore, we constructed a *hPIG-B* gene with *Drosophila* codon usage. This gene was expressed at a comparable level to hPIG-B with human codon usage in S2 cells, suggesting that the low level of expression is not due to differences in codon usage (data not shown). We then constructed *Drosophila*-based chimeric PIG-B proteins containing the human D2 and/or D4 subsegments, but found that they had no activity. We therefore constructed chimeric PIG-B proteins consisting mostly of *Drosophila* sequences, in which smaller regions were replaced with hPIG-B sequence (Fig. 5). In particular, we replaced the NE localization sequences of DmPIG-B with the corresponding portions of hPIG-B, and then examined whether the chimeric proteins localized to the ER in S2 cells and restored cell surface expression of uPAR in class B cells. One of the chimeric proteins, DmPIG-B[ER]5, was localized to the ER, and its expression level was comparable to that of DmPIG-B[NE] in S2 cells (Fig. 6A,B). DmPIG-B[ER]5 was detected in the ER, and not in the NE, in most cells, demonstrating that it primarily localizes to the ER. When expressed in CHO cells defective in endogenous PIG-B activity, DmPIG-B[ER]5 was localized to the ER and restored surface expression of uPAR (Fig. 6C,D), indicating that DmPIG-B[ER]5 represents the ER-localized functional form of the enzyme.

To express DmPIG-B[ER]5 and wild-type DmPIG-B[NE] at the same level, we generated upstream activating sequence (UAS) lines in which transgenes were inserted at the same chromosomal sites using the phiC31 system (Groth et al., 2004). We then expressed DmPIG-B[ER]5 and DmPIG-B[NE] in *PIG-B^13^* flies at 25°C using the daughterless (da)-Gal4 driver, which drives ubiquitous expression. Expression of DmPIG-B[NE] fully rescued the lethality of *PIG-B^13^*, whereas DmPIG-B[ER]5 did not (Fig. 6E). The mRNA levels of DmPIG-B[ER]5 and DmPIG-B[NE] were almost identical (Fig. 6F). By contrast, the amount of DmPIG-B[ER]5 protein was significantly lower than that of DmPIG-B[NE], as determined by immunoblot and immunofluorescence analyses (Fig. 6B,G). To compare the rescue efficiency between DmPIG-B[NE] and DmPIG-B[ER]5 at the same protein level, we decreased DmPIG-B[NE] expression to almost the same level as DmPIG-B[ER]5 by culturing flies at 18°C (Fig. 6B). Under these conditions, the rescue efficiency of DmPIG-B[NE] was still significantly higher than that of DmPIG-B[ER]5 (Fig. 6E), providing further evidence that NE localization of DmPIG-B is essential for its complete/optimal function.

DmPIG-B[ER]5 is a chimeric protein containing sequences of *Drosophila* and human, which are evolutionarily distant species. Thus, it is possible that this protein was misfolded in *Drosophila* cells. We compared the short sequences that were exchanged to create DmPIG-B[ER]5 between vertebrates and insects and found that their lengths clearly differ between these two groups (Fig. S3). To avoid any potential effects of this difference in length, we generated chimeric PIG-B proteins containing sequences of *Drosophila* and other insects. The chimeric PIG-B protein containing the ant sequence (DmPIG-B[ant]) was localized to the ER in S2 cells, whereas those containing the mosquito, bombyx and tribolium sequences (DmPIG-B[mos], DmPIG-B[bom] and DmPIG-B[tri], respectively) were localized to the NE (Fig. S4A). Assays of protein activity using class B cells revealed that all the chimeric PIG-Bs had full enzymatic activity (Fig. S4B). To determine whether the chimeric PIG-Bs function as efficiently as DmPIG-B[NE] *in vivo*, we performed rescue experiments as described above. NE-localized PIG-Bs (DmPIG-B[mos], DmPIG-B[bom] and DmPIG-B[tri]) rescued the lethality of *PIG-B^13^*, whereas the ER-localized PIG-B (DmPIG-B[ant]) did not efficiently rescue this lethality (Fig. S4C). Accordingly, expression of the ER-localized PIG-B was lower than that of the NE-localized PIG-Bs (Fig. S4D). These results are the same as those obtained using DmPIG-B[ER]5, as described above (Fig. 6) and support the notion that NE localization of PIG-B is essential for its optimal function, although the possibility that DmPIG-B[ant] and DmPIG-B[ER]5 were misfolded cannot be completely excluded.

Protein expression of DmPIG-B[ER]5 was markedly lower than that of DmPIG-B[NE]. We therefore investigated whether DmPIG-B[ER]5 protein was preferentially degraded. When wing disc cells expressing DmPIG-B[ER]5 were cultured in the presence of the lysosomal inhibitor bafilomycin A1 (Mauvezin et al., 2014), lysosomes were impaired and DmPIG-B[ER]5 protein was detectable (Fig. 6H,G). By contrast, when cells were cultured in the presence of the proteasome inhibitor MG132, ubiquitylated proteins accumulated, but DmPIG-B[ER]5 protein was not detected (Fig. 6I,G). These results indicate that DmPIG-B[ER]5 is preferentially degraded by lysosomes and not by the ubiquitin-proteasome system (UPS).

### NE localization of DmPIG-B may participate in the formation of a GPI modification zone

**Fig. 7.**
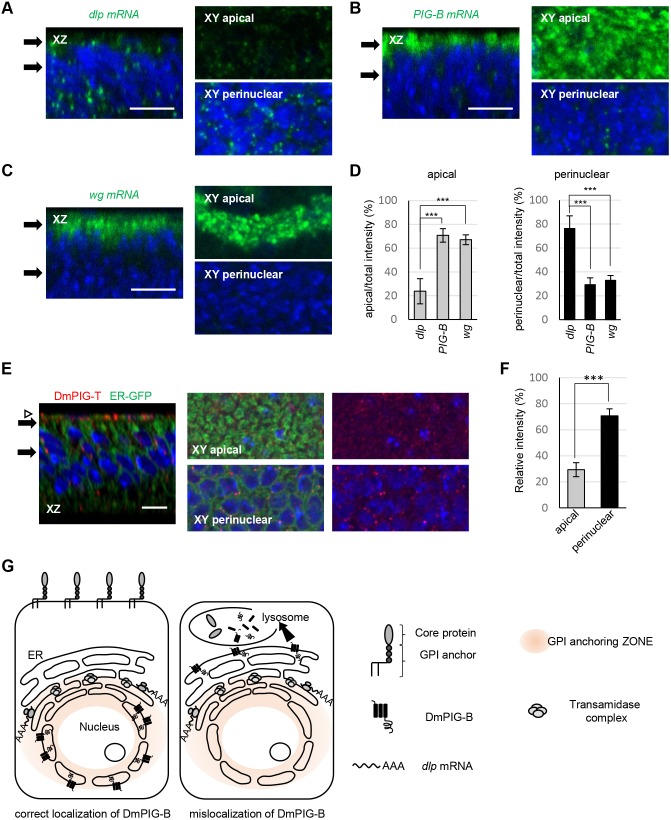
**Schematic representation of a functional platform (zone) for efficient formation of GPI-anchored proteins.** (A–C) Localization of *dlp* (A), *PIG-B* (B) and *wg* (C) mRNA (green) in wing discs. *xz* section (left), and apical (right upper) and perinuclear sections (right lower) of the *xy* plane, are shown. Black arrows indicate sectioning points. Peripodial membranes were removed in A and B after staining. Note that *dlp* mRNA is preferentially localized to the perinuclear region, whereas *PIG-B* and *wg* mRNA are localized to the apical regions. The nucleus is blue. Scale bars: 10 µm. (D) Statistical analyses of mRNA localization. Signal strength was calculated for each mRNA in the apical or perinuclear region. The ratio of the apical or perinuclear signal to the total signal is shown. Values are means±s.d. of data obtained in at least three wing discs. ***P<0.005 as determined by Student's *t*-test. (E) Localization of DmPIG-T (red) in wing discs expressing ER-GFP (green). *xz* section (left), and apical (middle and right upper) and perinuclear sections (middle and right lower) of the *xy* plane, are shown. Black arrows indicate sectioning points. The white arrowhead indicates peripodial membranes. The nucleus is blue. Scale bar: 10 µm. (F) Statistical analysis of DmPIG-T localization. Signal strength was calculated in the apical or perinuclear region. The ratio of the apical or perinuclear signal to the total signal is shown. Values are means±s.d. of data obtained in five wing discs. ***P<0.005 as determined by Student's *t*-test. (G) GPI synthesis, translation and GPI anchoring of GPI-anchored proteins occur in the NE and proximal ER (left, correct localization of DmPIG-B). Disorganization of the zone (e.g. owing to ER localization of DmPIG-B) results in degradation of GPI-anchored proteins and mislocalized enzymes in lysosomes (right, mislocalization of DmPIG-B).

To rationalize the NE localization of DmPIG-B, we sought to determine the ER site where other events in GPI-anchored protein production occur. We first determined where Dlp, a GPI-anchored protein, was produced by observing the localization of *Dlp* mRNA. *Dlp* mRNA was localized at the ER region proximal to the NE (Fig. 7A,D). To determine whether Dlp was selectively produced at the ER region proximal to the NE, we additionally observed the localizations of mRNAs encoding DmPIG-B, a transmembrane protein, and Wingless (Wg), a secretory protein. *PIG-B* and *wg* mRNAs were localized to apical regions in wing disc cells (Fig. 7B–D). Taken together, these results demonstrate that Dlp is selectively produced in the ER near the NE and/or in the NE. Dlp is cleaved and transferred to the GPI by the TAC. Hence, we generated an antibody against DmPIG-T, a component of TAC, and used it to stain wing discs. Immunofluorescence analysis revealed that DmPIG-T was also localized at the ER region proximal to the NE (Fig. 7E,F), while the ER was distributed throughout the entire cytoplasm (Fig. 7E). DmPIG-B and TAC were localized to the NE and perinuclear region, respectively; therefore, we investigated where the intermediate reactions between those catalyzed by DmPIG-B and the TAC occurred (Fig. 1A). PIG-F and PIG-O catalyze these intermediate reactions; therefore, we generated antibodies against DmPIG-F and DmPIG-O. Only the anti-DmPIG-O antibody recognized the endogenous protein. Immunostaining of ML-DmD9 cells with this antibody showed strong punctate signals proximal to the NE and weak signals in the entire extranuclear region (Fig. S5), suggesting that DmPIG-O forms large assemblies near to the NE. The signals disappeared when DmPIG-O was knocked down. These data suggest that translation, late steps of GPI synthesis and GPI-anchor formation for GPI-anchored proteins occur in the NE and the perinuclear ER. In summary, our findings suggest that the NE and the ER proximal to the NE provide a functional platform for efficient formation of GPI-anchored proteins by spatially juxtaposing late steps of GPI synthesis, translation and GPI anchoring (Fig. 7G).

## DISCUSSION

Synthesis of GPI-anchored proteins involves multiple biochemical reactions. Our results show that a late step of GPI synthesis conducted by DmPIG-B takes place in the NE, and that NE localization is essential for the function and stability of DmPIG-B.

NE localization sequences have been determined for several proteins, including the SUN (Sad1-UNC-84 homology) proteins, KASH (Klarsicht, ANC-1 and Syne homology) proteins, LEM (LAP2, emerin and MAN1) domain proteins and LBR (Barton et al., 2015; Hieda, 2017; Olins et al., 2010), but are not present in DmPIG-B. NCBI database searches for sequences that are homologous to the NE localization regions of DmPIG-B identified no known NE-localized proteins (data not shown). The NE localization sequences of DmPIG-B are distributed in four interspersed regions, which may form a structure that determines NE localization. Thus, a novel machinery may be responsible for localizing DmPIG-B to the NE.

Comparison of the short sequences that mediated the localization of vertebrate and insect PIG-B showed that their lengths differ between these two groups (Fig. S3). It remains possible that DmPIG-B[ER]5, a chimeric protein containing *Drosophila* and human sequences was misfolded because of this difference in length. Chimeric PIG-Bs containing *Drosophila* and human or ant sequences were localized to the ER, whereas those containing *Drosophila* and mosquito, bombyx or tribolium sequences were localized to the NE (Fig. S4A). In addition, all these chimeric proteins were fully active when assayed using class B cells (Fig. S4B). These results support the notion that the ER localization and instability of chimeric PIG-B[ER]s are not due to misfolding. Moreover, the sequences conserved in the NE-localized PIG-Bs suggest that WS(R/I)(K/R)Axxxx(L/I)(W/Y)xxA(L/A) is important for the NE localization of PIG-B (Fig. S3).

hPIG-B and DmPIG-B[ER] were barely detectable in *Drosophila* wing disc cells. One possible explanation is that a novel factor places DmPIG-B into the NE via the NE localization regions of DmPIG-B. Alternatively (or in addition), DmPIG-B may somehow be excluded from the ER. The latter possibility is supported by the observation that DmPIG-B[ER] was detected when lysosomal function was inhibited (Fig. 6G). The constitutive degradation of DmPIG-B[ER] suggests that it is recognized by the quality control machinery responsible for disposing of mislocalized proteins. It has been reported that mislocalized mitochondrial proteins that are misfolded without correct disulfide bonds are degraded by the UPS (Wrobel et al., 2015). By contrast, DmPIG-B[ER] is degraded by lysosomes and correctly folded. Thus, DmPIG-B may be degraded by a novel quality control mechanism in which DmPIG-B tethered to the NE is recognized as functional, whereas mislocalized DmPIG-B is sensed by an ER degradation system.

Next, we sought to explain why NE localization is required for DmPIG-B function. Our findings showing that *Dlp* mRNA and DmPIG-T were localized close to the NE (Fig. 7A–F) suggest that the ER proximal to the NE is the site of translation and GPI anchoring of GPI-anchored proteins, such as Dlp. Notably in this regard, in yeast, GPI-anchored proteins are loaded into transport vesicles distinct from those containing other transmembrane proteins (Muñiz et al., 2001). In addition, DmPIG-O, which catalyzes a reaction intermediate to those catalyzed by DmPIG-B and the TAC, was localized to the ER that was proximal to the NE (Fig. S5). Thus, our findings suggest that the NE and proximal ER provide a functional platform for efficient formation of GPI-anchored proteins by spatially juxtaposing late steps of GPI synthesis, translation, GPI anchoring and vesicle loading. We hereafter designate such functional platforms within an organelle or spanning multiple organelles as ‘functional organelle zones’ (Fig. 7G). Golgi units that undergo distinct types of glycosylation are another example of organelle zones (Yano et al., 2005). In addition, highly mobile structures in the ER may be another zone where phosphatidylinositol is synthesized and delivered to other organelles (Harayama and Riezman, 2018; Kim et al., 2011). In mammalian cells, some endogenous PIG activities are segregated into a particular ER subcompartment (Vidugiriene et al., 1999). However, PIGs overexpressed in mammalian cultured cells are uniformly localized to the ER. This discrepancy may arise because overexpression leads to production of excess protein that is then distributed throughout the ER. Because the GPI zone where GPI synthesis and anchoring occur in fly cells may be conserved in mammals, future work should seek to detect endogenous mammalian PIGs *in vivo*.

## MATERIALS AND METHODS

### Fly stocks

*Drosophila melanogaster* Canton-S (CS) was used as the wild-type strain. da-Gal4 was obtained from the Bloomington *Drosophila* Stock Center (BDSC). ER-GFP (CA06507) was a gift from Dr Allan C. Spradling (Department of Embryology, Carnegie Institution for Science, USA).

### Plasmid construction and generation of transgenic flies

To construct expression plasmids for Myc-tagged PIG proteins in S2 cells, cDNAs encoding DmPIGs were obtained from the *Drosophila* Genomic Resource Center (DGRC) (DmPIG-B; LD47795) (DmPIG-L; GM02889) (DmPIG-M; GH02741) (DmPIG-V; RE16378) (DmPIG-X; IP05792) (DmPIG-N; AT21454) (DmPIG-F; AT13969) (DmPIG-O; SD07983). A DNA fragment encoding the 3Myc sequence was ligated to the 3′ ends of the PCR-amplified coding regions of DmPIG cDNAs and inserted into expression vector pRmHa. To construct the expression plasmid for Myc-tagged human PIG-B, cDNA tagged with the 3Myc epitope at the N-terminus was cloned into pRmHa. To generate chimeric PIG-B expression plasmids, different gene fragments amplified from DmPIG-B and human, ant, mosquito, bombyx or tribolium PIG-B were fused using the in-Fusion HD cloning kit (TAKARA) and inserted into pRmHa. To construct the expression plasmid containing Calr–GFP, the Calr–GFP gene fragment was amplified from ER–GFP (CA06507) fly cDNA and inserted into pRmHa. To construct expression plasmids in CHO cells, PCR-amplified fragments were inserted into expression vector pCMVTNT (Promega). To generate transgenic flies, PCR-amplified fragments were cloned into pJFRC4-3xUAS-IVS-mCD8::GFP. Plasmids were injected into *y*^1^ *w*^67^*c*^23^; P{CaryP}attP2 embryos and integrated into genomic site 68A4 using the phiC31 integrase system.

### Generation of antibodies

Rabbit polyclonal anti-DmPIG-B and rat polyclonal anti-DmPIG-T antibodies were generated against synthetic peptides corresponding to the C-terminal 17 residues (AFNRGPDSGQHEPDVHD) of DmPIG-B and residues 609–625 (ISPGSPSGDQPLLEDLD) of DmPIG-T, respectively. A rat polyclonal anti-DmPIG-O antibody was generated against three synthetic peptides corresponding to residues 49–65 (AEYVLTDEVVNEIFKDV), residues 196–208 (RSYSYPSFDIFDL) and the C-terminal 19 residues (RIHRGVDTLIKRINKAKVH).

### Immunostaining

The following primary antibodies were used: rabbit anti-Myc A14 (1:500; sc-789, Santa Cruz Biotechnology), rabbit anti-Rab7 (1:500; Tanaka and Nakamura, 2008), mouse anti-lamin ADL67.10 [1:500; Developmental Studies Hybridoma Bank (DSHB)], mouse anti-Dlp 13G8 (1:500; DSHB), mouse anti-KDEL (1:500; SPA-827, Stressgen), and mouse anti-ubiquitin FK2 (1:1000; Cay14220, BIOMOL). For immunostaining, affinity purified rabbit anti-DmPIG-B antibody, affinity purified rat anti-DmPIG-T antibody and rat anti-DmPIG-O antibody were diluted at 1:5000, 1:5000 and 1:100, respectively. Alexa Fluor 488-labeled FLAER was obtained from CEDARLANE and used at 1 µM. LysoTracker Red DND-99 was purchased from Thermo Fisher Scientific and used at a concentration of 0.5 µM. For immunofluorescence experiments, wing discs dissected from *Drosophila* third-instar larvae, S2 cells, and CHO cells were fixed in 4% paraformaldehyde in PBS, blocked in 0.5% bovine serum albumin in 0.1% Triton X-100 in PBS, and then incubated with the appropriate primary antibodies. The specimens were then stained with the Alexa Fluor 488- (Thermo) or Cy3- (Millipore) conjugated secondary antibodies (1:500) and DAPI. Fluorescence images were captured under a laser-scanning confocal microscope (LSM710, Zeiss). The signal intensity of DmPIG-T was quantified in the apical or perinuclear region. The otal intensity was normalized by the region volume in which the signal was obtained. Relative intensity was calculated as below. Relative intensity [apical] (%)=normalized apical intensity/(normalized apical intensity+normalized perinuclear intensity)×100. Relative intensity [perinuclear] (%)=normalized perinuclear intensity/(normalized apical intensity+normalized perinuclear intensity)×100.

### Immunoelectron microscopy

Wing discs dissected from CS third-instar larvae were fixed with 4% periodate-lysine paraformaldehyde solution. Fixed discs were blocked in 5% normal goat serum in PBS and incubated with rabbit anti-DmPIG-B antibody (1:20), followed by incubation with horseradish peroxidase (HRP)-labeled anti-rat IgG (Jackson ImmunoResearch). After rinsing with PBS, they were fixed with 0.5% glutaraldehyde in PBS, washed with PBS, incubated with 0.025% 3.3′-diaminobenzidine tetrahydrochloride in 50 mM Tris-HCl (DAB solution), and incubated again in DAB solution containing 0.005% H_2_O_2_. The DAB reaction was stopped by rinsing the specimen with PBS several times. Finally, samples were treated with 2% OsO_4_ in 0.1 M phosphate buffer (pH 7.4), dehydrated with ethanol and embedded in Epon. Thin sections were made and examined on a JEM 1010 electron microscope.

### Generation of the *PIG-B* mutant

The P-element {GSV6}GS12796 obtained from the BDSC was mobilized to generate a null mutant allele by crossing with flies harboring a transposase (delta 2-3). About 100 white-eyed lines were screened by PCR for a deletion in the *PIG-B* coding region. The primers used for the screen were as follows: forward, 5′-TGCATATCCTAACACCTTAC-3′ and reverse, 5′-CGGAGTCACTATCAACTGGC-3′. Deletion lines were sequenced to determine the breakpoints.

### Cell culture and transfection

S2 cells originating from *D. melanogaster* were cultured in Schneider's medium supplemented with 10% fetal calf serum (FCS). ML-DmD9 cells originating from a wing disc of *D. melanogaster* were purchased from DGRC and cultured in M3 medium supplemented with 5% FCS, 0.05% KHCO_3_, 0.1% yeast extract and 0.25% bactopeptone (BPYE) and 10 µg/ml insulin. DNA transfection into these cells was performed with the calcium phosphate transfection reagent (Thermo). Protein expression was induced by incubating cells in the presence of 0.1 mM CuSO_4_ for 6 h. To knock down DmPIG-O, double-stranded RNAs corresponding to nucleotides 2637–2889 of DmPIG-O and the entire region of GFP (as a control) were synthesized using a T7 *in vitro* transcription kit (TAKARA) and transfected into ML-DmD9 cells.

The *PIG-B*-deficient cell line (CHO1.33) was generated by chemical mutagenesis of the parental CHO F21 cells and selection using the GPI-anchor-binding toxin aerolysin (Ashida et al., 2005). The *PIG-B*-deficient cell line (CHO1.33) is referred to as class B cells in the text. CHO-K1 cell lines (F21.3.8) stably expressing human CD59 and DAF, as well as class B cells were cultured in Ham's F-12 medium supplemented with 10% fetal calf serum, 600 µg/ml G418 and 400 µg/ml hygromycin. Plasmids were transfected into cells using Lipofectamine 2000 (Thermo).

### FACS

Surface expression levels of uPAR were determined by staining with anti-Chinese hamster uPAR (5D6) antibody (1:250, produced in the Kinoshita laboratory). Cells were stained with phycoerythrin-conjugated goat anti-mouse-IgG antibody (Becton Dickinson) and analyzed by flow cytometry (FACSVerse; Becton Dickinson).

### Immunoblotting

Homogenates of S2 cells, CHO cells or five larvae or adult files expressing DmPIG-B[NE], chimeric PIG-Bs or hPIG-B were subjected to SDS-PAGE. The proteins were transferred to PVDF membranes (Millipore), which were blocked with PBS containing 0.05% Tween 20 and 5% skimmed milk, followed by overnight incubation with the rabbit anti-Myc A14 (1:2000, sc-789, Santa Cruz Biotechnology) and rat anti-tubulin (1:2000, MCA78G, Oxford Biotechnology) primary antibodies. Primary antibodies were detected with alkaline phosphatase-conjugated anti-rabbit-IgG or anti-rat-IgG antibodies (1:10,000, Jackson ImmunoResearch), and the signals were visualized with the DDAO-phosphate substrate (Thermo). Images were acquired on a Typhoon9500 (Applied Biosystems).

### qPCR

Ten larvae were frozen at −80°C and then ground up. Total RNA was extracted using the RNeasy kit (Qiagen). Superscript reverse transcriptase (Invitrogen) and oligo(dT) primers were used for the reverse transcription reaction. Real-time quantitative PCR (qPCR) was performed on a 7500 HT Fast Real-Time PCR system (Applied Biosystems) with Power SYBR Green (Applied Biosystems). The amount of amplified transcript was normalized against an internal control (*rpl32*). The primers used for detection of Myc-tagged *DmPIG-B[NE]* and *[ER]* were as follows: forward, 5′-GAATCTGTTCAATGGGAGCTCC-3′; reverse, 5′-TGTCCAATTATGTCACACCACA-3′. The primers used for detection of *rpl32* are as follows: forward, 5′-GCAAGCCCAAGGGTATCGA-3′; reverse, 5′-CGATGTTGGGCATCAGATACTG-3′.

### Rescue experiment

*PIG-B*^13^ virgin females harboring da-Gal4 were crossed with *PIG-B*^13^ males harboring UAS-DmPIG-B as follows: *PIG-B*^13^ da-Gal4/TM6c × *PIG-B*^13^/TM6c, *PIG-B*^13^ da-Gal4/TM6c × *PIG-B*^13^ UAS-DmPIG-B[NE]/TM6c, *PIG-B*^13^ da-Gal4/TM6c × *PIG-B*^13^ UAS-DmPIG-B[ER]5/TM6c, *PIG-B*^13^, da-Gal4/TM6c × *PIG-B*^13^ UAS-hPIG-B/TM6c, *PIG-B*^13^ da-Gal4/TM6c × *PIG-B*^13^ UAS-DmPIG-B[ant, mosquito, bombyx or tribolium]/TM6c.

The offspring were cultured at 25°C or 18°C. The number of homozygous (*PIG-B*^13^/*PIG-B*^13^) and heterozygous (*PIG-B*^13^/TM6c) adult flies was counted, and the homozygote:heterozygote ratio was calculated.

### Bafilomycin and MG132 treatment

Wing discs dissected from third-instar larvae expressing DmPIG-B[NE] and DmPIG-B[ER]5 were incubated with M3 medium supplemented with 2% FCS, 2.5% fly extract and 10 µg/ml insulin in the presence of 100 µM bafilomycin, 10 µM MG132 or DMSO for 6 h. The discs were then processed for immunostaining.

### *In situ* hybridization

cRNA probes were prepared by subcloning of appropriate fragments from *PIG-B* cDNA clone LD47795, *wg* cDNA clone RE02607 and *Dlp* cDNA clone LD47466. The coding region of *PIG-B*, *wg* and four DNA fragments from *Dlp-RA* corresponding to nt 36–710, 711–1386, 1418–1898 and 2016–2298) were amplified by PCR and inserted into vector pCRTOPOII (Thermo). After linearization of plasmids, DIG-labeled RNA probes for *PIG-B*, *wg* and *Dlp* were generated using DIG RNA labeling mix (Roche). Wing discs dissected from CS third-instar larvae were fixed in 4% paraformaldehyde in PBS. After proteinase K treatment and post fixation, the specimens were pre-hybridized at 55°C with 50% formamide, 5× SSC, 100 µg/ml heparin, 100 µg/ml sonicated salmon sperm DNA and 0.1% Tween 20 (hybridization solution). The pre-hybridization solution was removed, and the specimens were incubated with probe solution (100 ng of probe in 100 µl of hybridization solution) at 55°C overnight. After washing, the specimens were incubated with HRP-labeled anti-DIG antibody in PBS containing 0.1% Tween-20 and 1% milk powder. Signals were developed using the Tyramide signal amplification kit (Thermo). DNA was detected by TOPRO3 staining. Fluorescence images were acquired on a laser-scanning confocal microscope (LSM710, Zeiss). The signal intensity of each mRNA was quantified in the apical or perinuclear region. Total intensity was normalized by region volume in which the signal was obtained. Relative intensity was calculated as below. Relative intensity [apical] (%)=normalized apical intensity/(normalized apical intensity+normalized perinuclear intensity)×100. Relative intensity [perinuclear] (%)=normalized perinuclear intensity/(normalized apical intensity+normalized perinuclear intensity)×100.

## Supplementary Material

Supplementary information
