# Supplementary material for: Nuclear envelope localization of PIG-B is essential for GPI-anchor synthesis in Drosophila
Source: J Cell Sci. 2018 Oct 26;131(20):jcs218024. doi: 10.1242/jcs.218024 (PMC6215393; doi:10.1242/jcs.218024)
Supplement: Supplementary information [file joces-131-218024-s1.pdf]

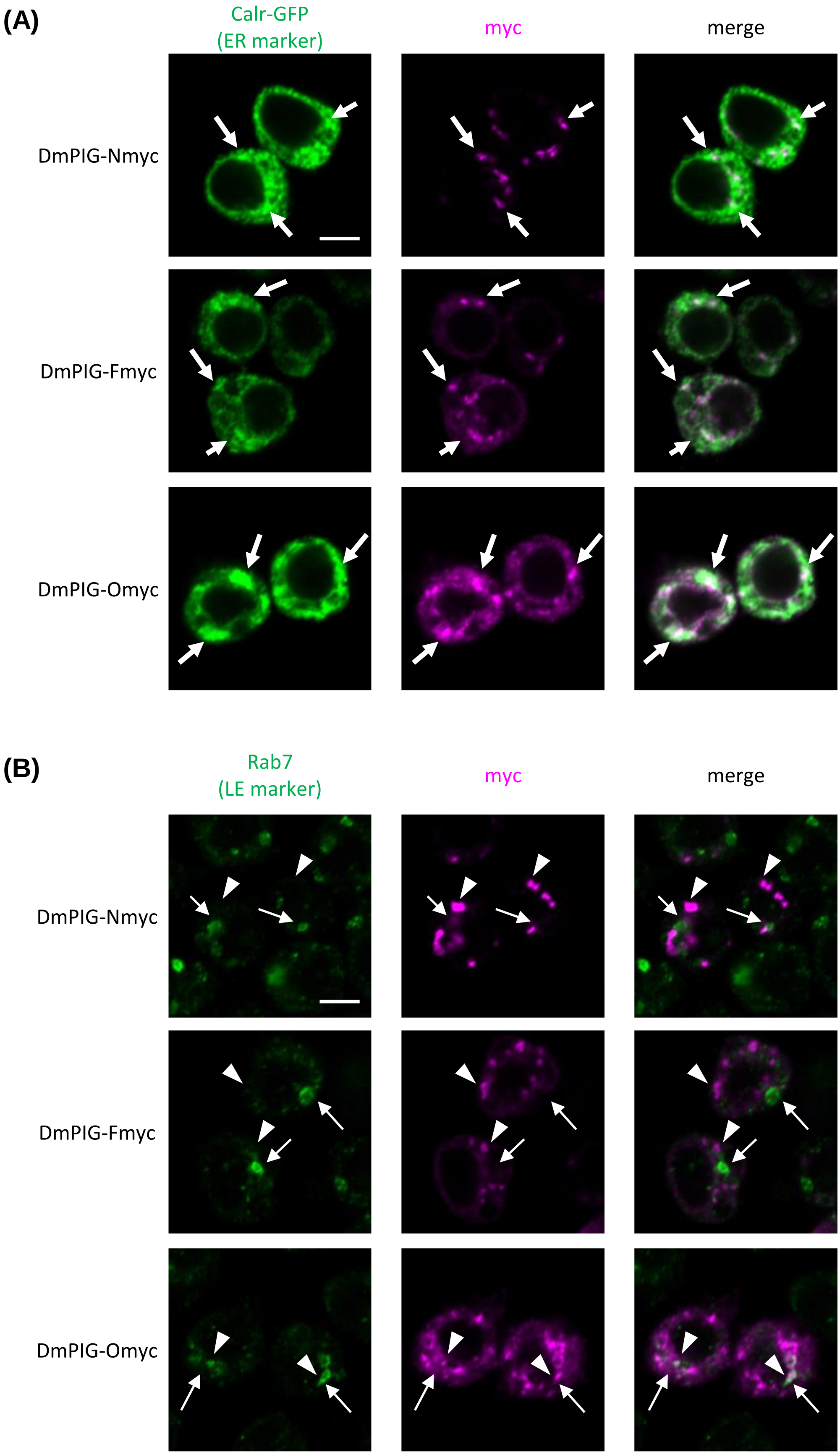

Journal of Cell Science • Supplementary information

Fig. S1. Co-staining for organelle markers and myc-tagged DmPIG-N, -F, or -O in S2 cells

(A) Immunofluorescence analysis of DmPIG proteins and Calreticulin (Calr-GFP, ER marker) in S2 cells expressing myc-tagged DmPIG proteins (DmPIG-N, -F, and -O) and Calr-GFP. Myc-tagged DmPIG proteins were stained with anti-myc antibody (magenta) and the ER was labeled with anti-GFP antibody (green). Arrows indicate co-localization of DmPIG proteins with Calr-GFP. Bar, 5 μm.

(B) Immunofluorescence analysis of DmPIG proteins and Rab7 (late endosome marker) in S2 cells expressing myc-tagged DmPIG proteins (DmPIG-N, -F, and -O). Myc-tagged DmPIG proteins were stained with anti-myc antibody (magenta) and late endosomes were labeled with anti-Rab7 antibody (green). Arrows indicate Rab7-positive endosomes and arrowheads indicate DmPIG proteins. Bar, 5 μm.

Journal of Cell Science • Supplementary information

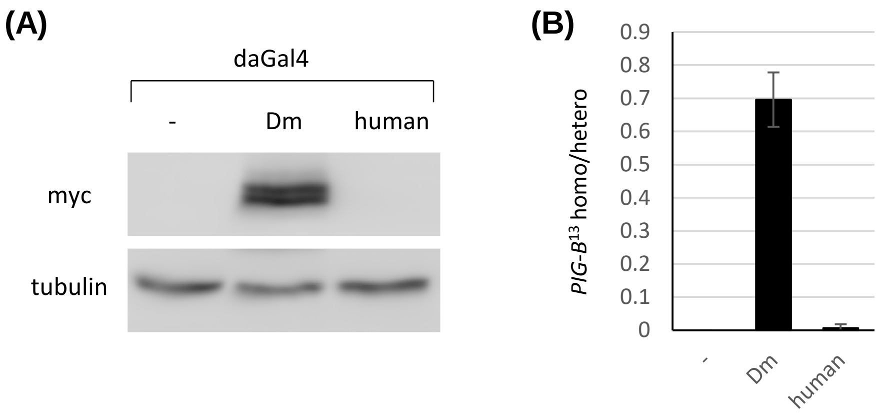

Fig. S2. Low expression level and rescue efficiency of human PIG-B in *Drosophila*

(A) Immunoblot analysis of myc-tagged DmPIG-B (Dm) and human PIG-B (human) expressed in third instar larvae under the control of da-Gal4. Proteins were detected with anti-myc and anti-tubulin (loading control) antibodies.

(B) Rescue of *PIG-B*^{13} lethality by expression of myc-tagged DmPIG-B (Dm) and human PIG-B (human) under the control of da-Gal4 at 25° C. Values are means ± S.D. of data obtained in three independent crosses; more than 100 adults were scored for each cross.

Journal of Cell Science • Supplementary information

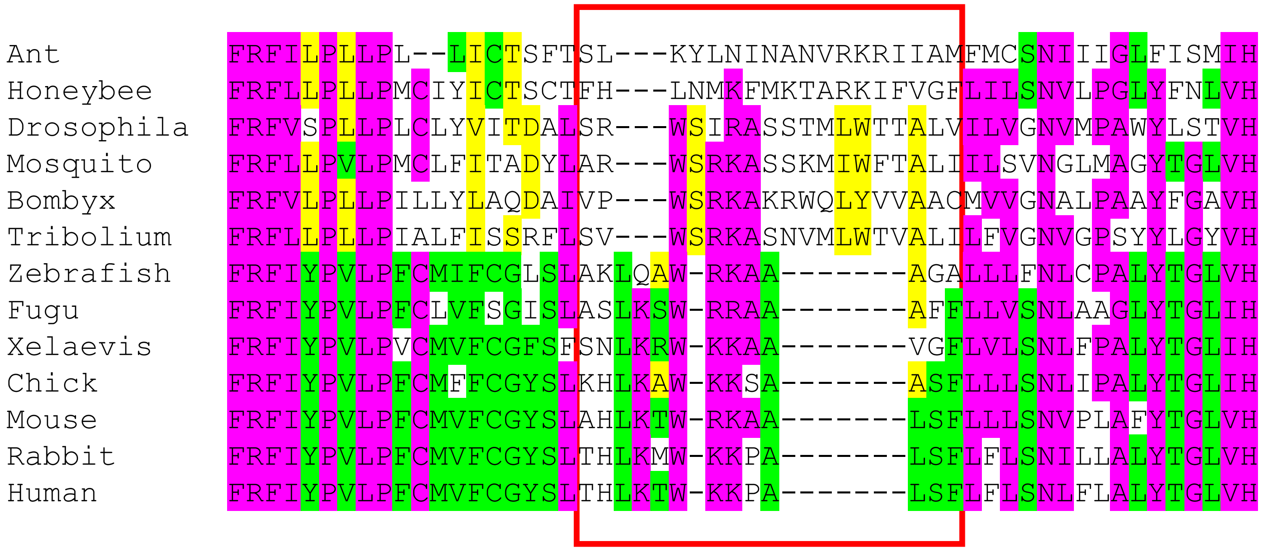

Fig. S3. Comparison of the amino acid sequences of PIG-B in various organisms

Amino acid sequences of D4 segment of *Drosophila* and the corresponding segments of *Atta cephalotes prog* (ant), *Apis mellifera* (honeybee), *Anopheles gambiae* (mosquito), *Bombyx mori* (bombyx), *Tribolium castanet* (tribolium), zebrafish, fugu, Xenopus, chick, mouse, rabbit, and human were aligned. Amino acids conserved in most species, insects, and vertebrates are shown in red, yellow, and green, respectively. The corresponding regions exchanged between *Drosophila* and human, ant, mosquito, bombyx, and tribolium are shown in a red box.

Journal of Cell Science • Supplementary information

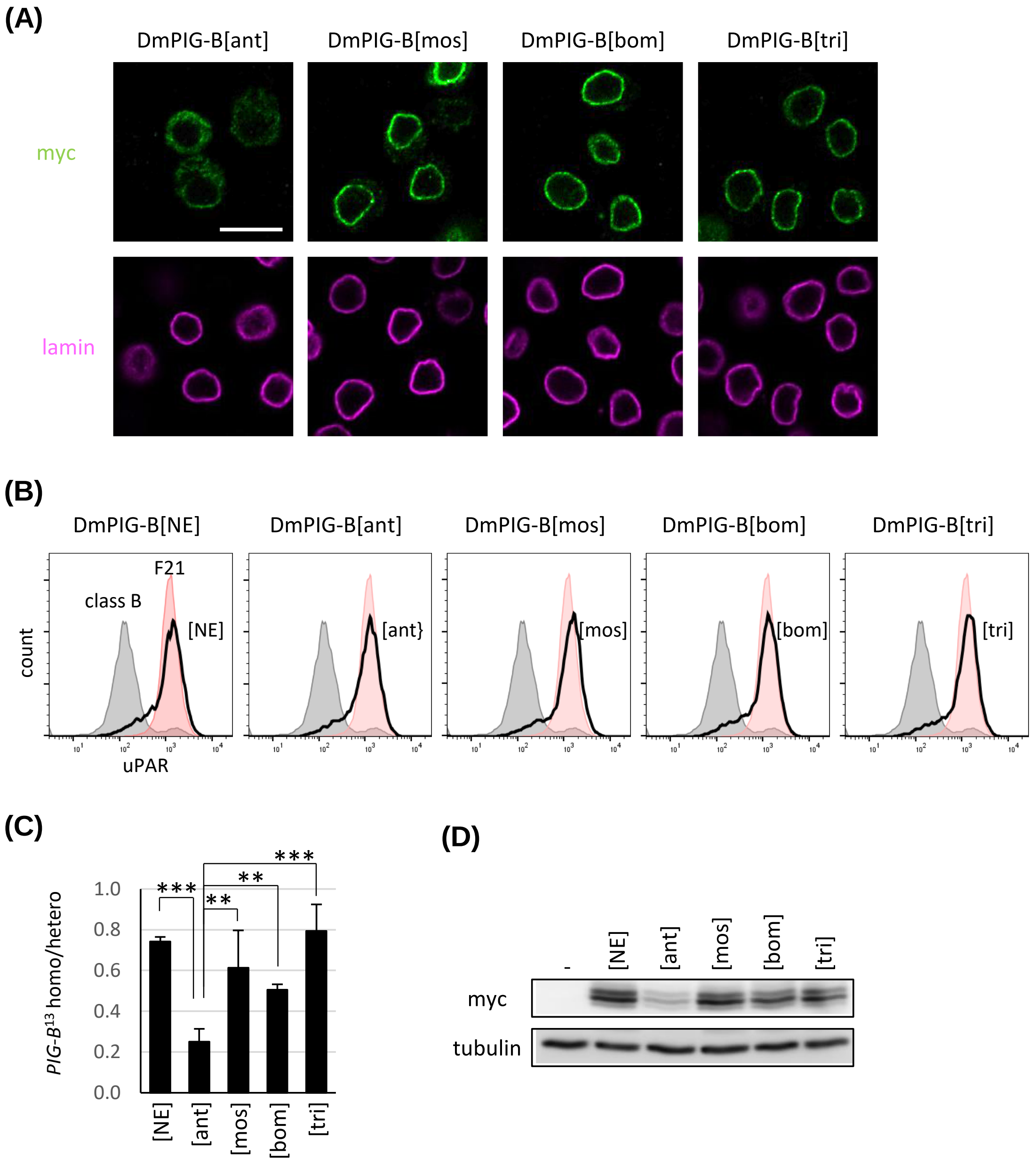

Fig. S4. Localization, activity, rescue efficiency, and expression level of chimeric PIG-Bs containing sequences of *Drosophila* and ant, mosquito, bombyx, or tribolium

(A) Immunofluorescence analysis of S2 cells expressing myc-tagged chimeric PIG-Bs containing sequences of *Drosophila* and other insects. Cells were stained with anti-myc (green) and anti-lamin (magenta) antibodies. Bar, 10 µm.

(B) Restoration of surface expression of uPAR on class B mutant CHO cells by transfection of chimeric PIG-Bs. Gray, class B cells; red, parental cell line F21; bold black line, class B cells expressing each chimeric PIG-B.

(C) Rescue of $\textit{PIG-B}^{13}$ lethality by expression of myc-tagged DmPIG-B[NE] and chimeric PIG-Bs under the control of da-Gal4 at 25° C. Values are means $\pm$ S.D. of data obtained in three independent crosses.

(D) Immunoblot analysis of myc-tagged DmPIG-B[NE] and chimeric PIG-Bs expressed in adult flies under the control of da-Gal4. Proteins were detected with anti-myc and anti-tubulin (loading control) antibodies.

Journal of Cell Science • Supplementary information

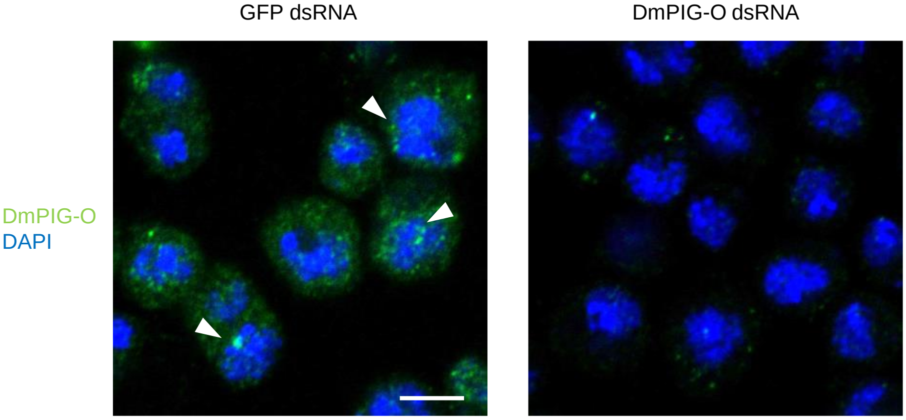

Fig. S5. Localization of endogenous DmPIG-O in ML-DmD9 cells
Immunofluorescence analysis of DmPIG-O in ML-DmD9 cells in which GFP (GFP dsRNA) or DmPIG-O (DmPIG-O dsRNA) was knocked down. Cells were stained with an anti-DmPIG-O antibody (green) and DAPI (blue). Arrowheads indicate strong punctate signals of DmPIG-O proximal to the NE. Bar, 5 µm.
